# Systemic Problem Structuring in a Complex Hospital Environment using Viable System Diagnosis – Keeping the Blood Flowing

**DOI:** 10.1007/s11213-021-09569-6

**Published:** 2021-04-24

**Authors:** Maren Berge Vik, Hanne Finnestrand, Robert L. Flood

**Affiliations:** Department of Industrial Economics and Technology Management, The Norwegian University of Science and Technology (NTNU), Trondheim, Norway

**Keywords:** Viable Systems Model (VSM), Viable System Diagnosis (VSD), Hospital, Systemic thinking, Complex organisations

## Abstract

This article presents the application of the systemic problem structuring approach Viable System Diagnosis (VSD) within the Department of Orthopedic Surgery in a large hospital in Norway. It explains why systemic thinking is relevant to this uniquely complex form of human organization. The department was coping with systemic dysfunction and VSD was chosen because previous applications demonstrated VSD excels at diagnosis of what is causing dysfunction. VSD was employed through a participatory framework that included in the process, among other stakeholders, medics, technologists, managers, administrators and, as far as possible given the sensitive nature of patient information, the patient. VSD guided thinking about what the organization is set up to do and the existing organizational arrangements to achieve that. The outcome was an agenda for debate that guided stakeholder discussions toward ways and means of improving organizational arrangements. The article briefly reviews previous applications of VSD in the hospital sector and other large complex organisations.

## Introduction

Hospitals in advanced economies face a substantial increase in chronic health problems and number of patients; the result of lifestyle-induced diseases and an overall longer life expectancy. Advances in medical knowledge and technology facilitate diagnosis and treatment of a wider range of illnesses and create even more demand on physical and human resources. In many cases the increase in demand outstrips the increase in resources so that delivering effective hospital care has become ever-more challenging and stressful for hospital staff.

There is a growing need in hospitals for problem structuring approaches that help hospital staff to gain a better understanding of the complex systemic nature of their work environment, thereby leading to systemic improvement and more effective and enjoyable work practices. Here, we report an application of the systemic problem structuring approach Viable System Diagnosis (VSD) to the care of orthopedic patients in a hospital in Norway. VSD focuses on diagnosing weaknesses in organizational arrangements. Our approach to VSD emphasises a participatory approach that engages involved and affected parties in a meaningful and fair process of identifying weaknesses and ways of tackling them.

### General Issues Faced by Hospitals

The structure of the hospital, the profession’s diversity, and the fundamental need for effective communication and action, embracing a wide range of stakeholders, all point to a uniquely complex form of human organization (Drucker 2006). The raison d’être of a hospital is to provide health care to patients and to this end it is organised into multiple clinics and wards to cater for numerous medical categories. Day-to-day life involves routine tasks interrupted by non-routine emergencies. A substantial part of everyday medical care is emergency patients and emergencies are unpredictable in timing and type. Medical activities are supported by a range of non-medical occupations, including general administration and specialists such as information scientists and economists. Hospitals are influenced by an attentive media and hospital care is high on the agenda of citizens and politicians alike. Wider socio-economic issues have a controlling effect. At the centre of it all, of course, is the patient and the patients’ needs are more than the treatment of an illness; they include mental health care, after care, family support and so on.

### The Department of Orthopedic Surgery

A few years ago, the Management of a large hospital in Norway established a cooperation agreement with our university. Their aim was to improve organizational efficiency and develop skills in change management. A joint post-doctoral position was created along with several additional collaborative research and development projects. Our project in the Department of Othopedic Surgery (DOS) addressed unsatisfactory waiting times for orthopedic patients.

Preliminary time spent with stakeholders of Department of Orthopedic Surgery in August 2017 and January 2018 revealed that its operations were dogged by inadequate communication between stakeholders, poor coordination of key healthcare activities, problems in information gathering and sharing, and intelligence-starved policy making and implementation. All staff were highly committed to providing health care, but the organizational arrangements prevented them from delivering the standard of healthcare to which they aspired. We proposed to the hospital’s Projects Management Team (Clinic Manager, Head of Nurses, and occasional co-opted personnel) the systemic process of learning and problem structuring known as Viable System Diagnosis and our proposal was well received.

### Why Systemic Thinking and Viable System Diagnosis for Hospitals?

Chapman (2002) warns health services away from reducing complex problems into separate so-called ‘manageable components’, since challenges in healthcare span those components. Mazzocato et al. (2010) provide evidence that hospitals as systemic arrangements are not adequately supported by reductionist methods. They argue that the focus of many organizational improvement initiatives is overly narrow with limited organizational reach. Healthcare organizations are better advised to work across traditional functional divides to create value for patients and other stakeholders. Systemic issues such as inadequate communication and coordination are frequently cited as major problem areas in the hospital sector (e.g., Coiera et al. 2002; Arora et al. 2005; Alvarez and Coiera 2006; Faraj and Xiao 2006). This is especially the case for patient groups that need coordinated services, such as patients with composite diseases and elderly people experiencing complex sufferings that require the attention of several specialists (Kirsebom et al. 2013; Roberge et al. 2016). Systemic failings in such areas featured strongly during our study and were the source of unacceptable waiting times for orthopaedic patients. There was a degree of systemic dysfunction in DOS. We found Viable System Diagnosis highly relevant because its systemic principles place emphasis on diagnosing what is causing dysfunction in organizations and avoids creating efficiency and effectiveness in one place that results in counter-intuitive inefficiencies and ineffectiveness in other places.

## Methods

Viable System Diagnosis (VSD) is a recognized problem structuring approach with many practical examples (e.g., Beer 1985; Espejo 1990; Espejo and Reyes 2011; Espinosa and Duque 2018; Espinosa et al. 2008; Harwood 2019). In our interpretation of VSD, the *purpose* is to diagnose an organization for effectiveness (i.e., effectiveness in achieving what it is set up to do). The *process* of VSD facilitates stakeholders thinking through what the organization is set up to do and the existing organizational arrangements to achieve that. The *outcome* of VSD is an agenda for debate that guides stakeholder discussions toward improving organizational arrangements. The *principles* of VSD systemically envision stakeholders (Hildbrand and Bodhanya 2014) as actors ‘involved in and affected by’ (Churchman 1971, 1979; Ulrich 1994), in our case, the process of treating orthopedic patients. Stakeholders with different roles and responsibilities tend to emphasize different issues (Guldbrandsen 2010). VSD as a participatory process helps to surface the perspectives of multiple stakeholders about the main organizational issues faced. In our case, stakeholders included nurses, surgeons, administrators, planners, staff holding leadership responsibilities, staff that did not, patients and so on.

VSD operationalizes a generic model of what constitutes a viable organization called the Viable System Model (VSM). The VSM represents a major part of the lifework of Stafford Beer (e.g., Beer 1972, 1979, 1985). Viability in essence means an ability to work successfully. VSD is introduced below through our case study.

**Fig. 1:**
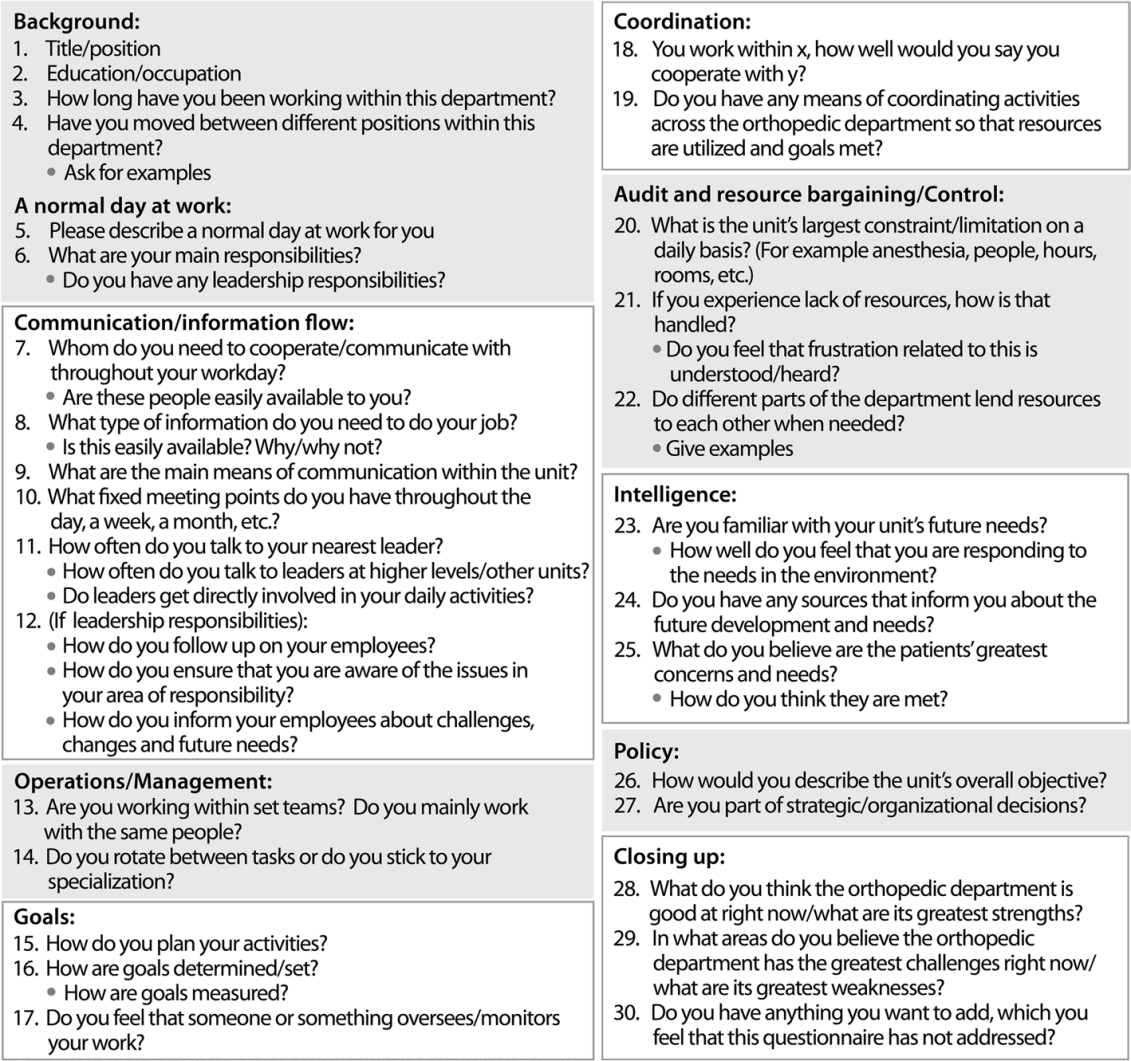
Semi-structured interview guide showing questions and categories

We facilitated VSD learning about the handling of orthopedic patients by: (1) ‘Shadowing’ the work activities of one of the key personnel for two days. (2) Issuance of an ID-card enabling our access to the hospital premises permitting general observation. (3) Facilitating qualitative semi-structured interviews (Fig. 1). (4) Attending management meetings and member-check meetings. (5) Holding regular meetings with the hospital’s Projects Management Team. Our aim was to identify stakeholders, to draw together their ‘different realities’ concerning the main organizational issues faced, and to structure these issues using VSD into a useful agenda for debate about improving the organizational arrangements.

‘Shadowing’ is a nonparticipant observation technique where the researcher follows an organizational member conducting their job. It enables a researcher to observe work activities without simultaneously having to act, and provides a unique way to gain first-hand knowledge without attaining a specific skillset through formal training (Czarniawska 2007). It was important to us ‘friendly outsiders’ (Greenwood and Levin 2007) to become thought of as ‘friendly insiders’ rather than some external stranger conducting research. ‘Member-check’ means that data, analytic categories, and interpretations are tested with members of the stakeholder groups from which the data was originally collected (Lincoln and Guba 1985).

It was not possible for us to freely decide who to interview due to the nature of the organization’s daily activities. Most employees could be absent from their tasks only for a limited time as most were directly involved in ongoing patient care. The project’s contact person within the clinic provided a list of personnel to approach, spread across the unit and with a range of leadership responsibilities. The list became a start-point in the search for other informants. As we progressed, we experienced a ‘snowball-effect’ and gained access to employees involved in daily operations. Input from leaders as well as employees with limited task responsibility is highly desirable. Other informants were approached that had specialist knowledge on topics that surfaced during interviews. There was limited access to patients. Ideally, patients would be interviewed to gain insights into their experiences as a client. However, direct access to patients was restricted due to their illnesses and the sensitivity of patient information. So, we made a special effort to ‘hear the patients’ voice’ when informants referred to patients.

Fourteen interviews were completed in Spring 2018. Six interviewees came from nursing-related activities and two had management responsibilities. Five interviewees came from surgeon-related activities; three were medical staff with management responsibilities, two were administrative staff, one with management responsibilities. The remaining interviews were carried out with support staff. The semi-structured interview was designed in accordance with the five categories/ ‘systems’ of VSM introduced below: operations, coordination, control, intelligence, and policy.

Responses to category specific questions inevitably crossed category ‘boundaries’ since organizational issues are of a systemic nature. So, responses were coded with the most relevant category/categories. The interview guide was tested and improved in three pilot interviews with personnel from similar organizational settings. Some of the interview questions were modified in the early stages of data collection based on further insights gained into the usefulness of the interview. Also, the first few interviews helped us to better appreciate areas for which more knowledge was needed and so questions were added to the interview guide (Hildbrand and Bodhanya 2015).

### Viable Systems and the Department of Orthopedic Surgery

VSM and VSD change how we look at management, traditionally operated as a hierarchical, top-down structure characterised by old-fashioned command and control (Herrera et al. 2011). Stafford Beer understood that hierarchical organizational charts as much as anything else are employed as a tool to apportion blame (Beer 1984; Flood 1999). Redistribution of decision-making ‘power’ is an important principle of VSM and VSD. Hierarchical organizational charts are set aside in favour of understanding organizational functions independently of who currently fulfils or executes them (Leonard 2009). With a picture of an ideal functionality comes the principle of redistribution of decision-making power to give sufficient autonomy and flexibility to functional parts so that they can maintain their own viability. Thus, a viable organization comprises several other viable organizations, all operating in cohesion through communication channels for coordination, control, intelligence, and policy. Ideal for viability is that organizational relations comprise only the relations that are necessary and sufficient for an organization to be able achieve what it is set up to do (Achterbergh and Vriens 2010). Viable systems are found within viable systems at increasing levels of complexity (Espejo and Reyes 2011). Such an arrangement is known as ‘recursion’ and is distinctly different from hierarchy. In a recursive organizational structure, any viable system contains and is contained within a viable system (Beer 1979). With recursion there is functional decentralisation and cohesion of the whole (Espejo and Gill 1997).

**Fig. 2:**
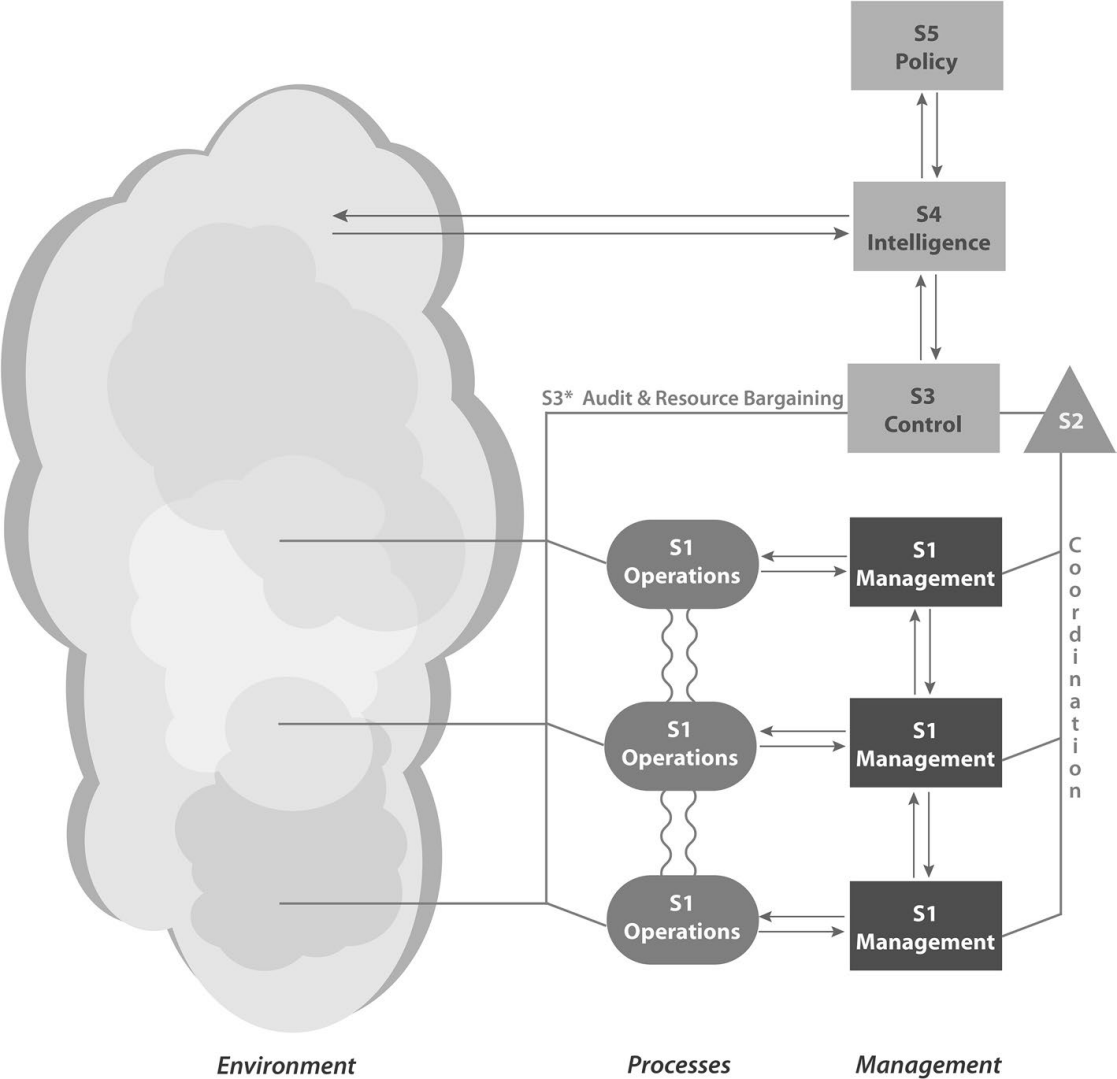
A basic depiction of the generic Viable System Model

The VSM can be depicted diagrammatically (Fig. 2). Beer used the terms ‘System 1′, ‘System 2′, … up to ‘System 5 to represent essential management functions. These terms are abstract and difficult to communicate to practitioners such as staff in a hospital. We replace them with everyday terms (Flood and Jackson 1991; Flood 1995) ‘Operations’ (System 1), ‘Coordination’ (System 2), ‘Control’ (System 3), ‘Intelligence’ (System 4), and ‘Policy’ (System 5). Purists of the VSM may find this hard to accept, but pragmatists find it essential. After all, the essence of VSM is to communicate.

#### Operations

**Fig. 3:**
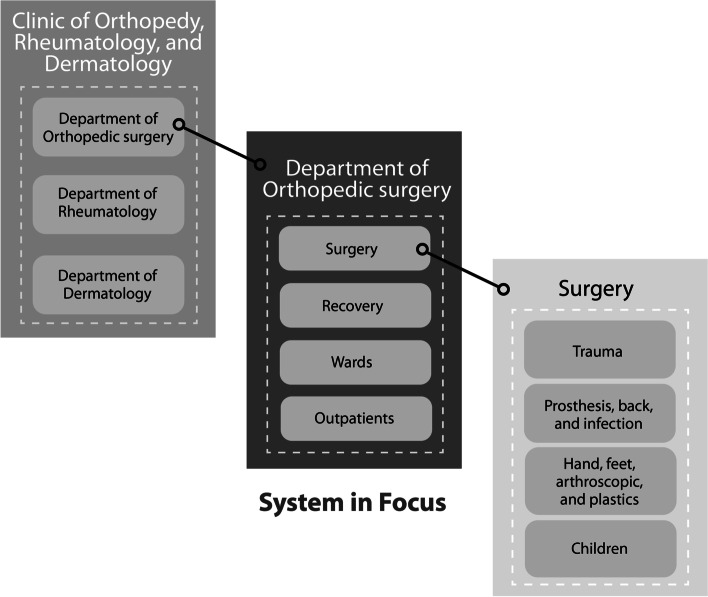
The system in focus in our study: the Department of Orthopedic Surgery

The purpose of an organization is what the organization does (Beer 1979). Operations fulfil the organization’s purpose (Espinosa and Walker 2013). VSD operationalizes the VSM and identifies the operations by asking, ‘What primary activities of the organization fulfil its purpose?’ Primary activities are organised into what we call ‘Divisions’. Each Division is defined by its Processes (depicted as an oval shape) and its Management (depicted as a rectangle). The common understanding at the hospital level is that Divisions are clinics, such as the Cancer Clinic, Children’s Clinic, Clinic of Cardiology and, in our case, the Clinic of Orthopedy, Rheumatology, and Dermatology (Fig. 3). Management of these clinics requires expertise about capacities and market requirements of the Processes (Leonard and Beer 1994). Our engagement at the hospital came about because staff of the clinic felt that something was wrong with capacities and satisfying market requirements.

Operations and its Divisions provide the organization’s core value-creating activities and are what the organization exists to do (Flood 1995). Operations communicate (lines depict communication) with each other. For example, in the Department of Orthopedic Surgery, the staff of Surgery, Recovery, Wards, and Outpatients must be in regular communication. Also, a Division must communicate with and provide a service to its Environment (depicted as an amoeba shape) (Leonard and Beer 1994). Broadly speaking, the Environment of a Division involves the external actors, activities, and issues that affect the Division. For example, a department in a hospital has a specific type of patient (customer) and may need specialist suppliers to resource the unique services that it provides to its patients. That said, some actors, activities, and issues may be relevant to several departments (depicted by overlapping Environments). For example, many departments require personal protective equipment for infection control. A principle of the VSM is that Divisions must have local autonomy, but only in so far as it does not compromise overall coherence of the organization. Local autonomy is necessary to carry out day-to-day activities effectively, to make decisions about them, and to adapt quickly to environmental change (Espinosa and Walker 2013). To this end, Divisions ultimately must themselves be Viable Systems (the principle of recursion mentioned above; Fig. 3; Beer 1979).

Divisions are not necessarily as obvious as clinics and departments in a hospital, and arguably a thorough diagnostic process should question even the obvious. That is why VSD begins by ‘recognizing’ Divisions from the complex of activities. The process defines the ‘System in Focus’ (activities in the organization that the study will focus on). The most focused System in Focus is concentrated on the most pressing issues that need attention. In our study, the hospital’s Projects Management Team was aware of hospital-wide issues through ongoing dialogue with a range of staff. Among other issues, they recognised particularly pressing issues in the waiting times of orthopedic patients. The Projects Management team invited us to work in the Department of Orthopedic Surgey and by this process the department became the System in Focus (Fig. 3). In general, Divisions of a System in Focus may represent geographical layout, activity type, required resources, client types and so on. Representing a hospital in terms of Divisions as clinics is an example of mapping a hospital’s operations onto client types.

Viability requires a Coordination function (depicted as a triangle) to support short-term activities of Divisions. This involves the coordination of available resources and managing conflict between Divisions. Coordination receives information about short-term changes and challenges in the Divisions and applies agreed procedures to deal with them. The stronger the coordination between Divisions, the greater the chance for synergy, and the less the need for management intervention. In order not to confuse the term ‘coordination’ with top-down management control, it is better to understand the process of coordination as, ‘coordination by mutual adjustment between support functions and between autonomous units’ (Espejo and Gill 1997). An example from our study is a scheduling system or a weekly status meeting with the purpose of discussing and balancing ward capacity in the System in Focus (Hildbrand and Bodhanya 2015).

The Control function is another management function (thus, depicted as a rectangle) that is responsible for longer term issues than those dealt with by Coordination, such as insufficient resources to meet growing demand. Issues outside the scope of the Coordination function should automatically activate control procedures (Flood 1995). Through ‘System 3*’, Control also handles resource bargaining and performs various routine audits by monitoring crucial variables that indicate the health of the System in Focus (Hildbrand and Bodhanya 2015). It carries out special audits of the Divisions *without intervening* in the Divisions (our emphasis; Leonard 2009). It verifies information given by Divisions (Achterbergh and Vriens 2010). An example of a routine audit in our department is monitoring the number of infections after surgery. An example of resource bargaining is biannual meetings to evaluate and disperse ward capacity to departments.

Resource bargaining is needed to facilitate the running of an organization in the best interests of the whole organization, not solely the best interests of the Divisions (Leonard 2009). It plans and ‘optimizes’ activities of Divisions so that the organization benefits from synergies that arise when the units work cohesively as part of the whole organization (Espinosa and Walker 2011). With information generated by System 3*, Control can reorientate behaviors that threaten organizational viability (Espinosa 2015).

The Intelligence function is a management function (depicted as a rectangle). In essence, Intelligence assesses the strengths and weaknesses of the organization (by well-defined communication with Control) and the opportunities and threats in the organization-wide Environment (depicted by a large amoeba shape that incorporates the Divisions’ Environments). The Organizational Environment also includes the future environment (Leonard and Beer 1994; Flood 1995). Intelligence builds scenarios about what the future might look like given the strengths, weaknesses, opportunities, and threats. Information/learning through Intelligence must be distributed to all Divisions that would benefit from or need to know about it. Intelligence provides the basis, for example, for recruitment and staff development in the Department of Orthopedic Surgery, among many other things.

The Policy function is a management function (depicted as a rectangle) and completes the basic arrangement of a VSM. Policy is responsible for strategy, mission, goals, objectives, values, and culture. Although unique procedures define Policy, all management functions ideally contribute, and Policy should not be interpreted as a top-down activity. One role of Policy is to modify policies of the System in Focus, based on relevant information reaching it, which in turn modify the procedures of the other management functions. What a viable system does is done by Operations; Policy, then, is ‘only’ thinking about what is done (Beer 1985).

Crucial to the VSM arrangement, hence viability, are the vertical and horizontal communication channels or flows of information (depicted by lines). In a hospital, or indeed any organization, communication more than anything else ‘keeps the blood flowing’. If information flow is poor, then, according to VSM principles, the organization is in poor health.

A viable system is part of a wider activity and policy modifications must be made in the context of the wider whole. As explained above, a viable system co-evolves with its environment by adapting to environmental changes. It needs to be autonomous so that it can quickly adapt to changes in the local environment but must also be able to keep a healthy relationship with the viable systems that it contains and the viable system that it is contained within (Espinosa and Walker 2011, p. 28).

The VSM is not in any sense an organizational chart with senior managers at the top. Turn it upside down and the meaning of what is described above remains unchanged. Turn an organizational chart upside down and the meaning is turned upside down, with ‘workers’ becoming top managers and senior managers becoming ‘workers’. Indeed, the VSM may be better understood upside down since fundamentally it is the role of management functions to enable/support the Divisions (Flood 1995).

The following results, reflections and questions offer critical insights into the organizational arrangements of the Department of Orthopedic Surgery and lead to the generation of an agenda for debate for stakeholders about improving the organizational arrangements.

## Results, Reflections and Questions

### Operations

As previously stated, what a System in Focus does is done by its Operations. The role of Operations in the VSM is to fulfil the purpose of the organization. VSD thus begins by identifying the primary activities of the organization as defined by its Divisions. Nurses and surgeons of the Department of Orthopedic Surgery are organized into four Divisions: Surgery, Recovery, Wards, and Outpatients. Each Division has its own Management support. Nurses are either ward nurses or theatre nurses.

In response to our questions, nurses and surgeons alike stated that the purpose or the value-creating activity of the department is patient treatment in accordance with health standards. To this end, staff work together to assess patients and to plan activities, from initial contact all the way through to outpatient services. Patients are dealt with in the wards before and after surgery by ward nurses and surgeons, while surgeons perform surgery and theatre nurses assist during surgery. On the face of it, the role and activities of surgeons and nurses in everyday operations is clear-cut. However, their experiences paint a different picture.

Surgeons move between activities during their working week, which is challenging in terms of planning and coordination and sometimes runs into task conflict:

I have two, three days where I do surgery. Then I have one day for the doctor’s round; usually I do the doctor’s round before noon and take care of paperwork after lunch. On a surgery day, I usually have two or three surgical interventions and I might also do a doctor’s round [visit patients in the wards] before that and some paperwork in between and after that. Then I usually have a day at the Outpatient Division. Surgeon with leadership responsibilities

Most ward nurses described their workday as eventful and full of frantic activity:

We have a hectic day from start to end. In the morning, there are often patients that need to be prepared for surgery. Then there is breakfast, medicines, looking after the patients, and the doctors’ round. Then you may have to plan discharge of patients. There are many tasks related to the patients and a lot of documentation. It is a hectic workplace. Even though it is unpredictable, you know what you need to do to get through the day. You never know what will happen, but still, there is a sense of system to it. Ward nurse

Several activities have more than one leader because they transcend occupational groups. In general, though, surgeons lead surgeons and nurses lead nurses. The responsibility of leaders was well stated by a theatre nurse:

As leaders in the hospital, we have three main areas of responsibility: profession, personnel, and economy. Theatre nurse with leadership responsibilities

Nurses with leadership responsibility are freed from operational duties to release time for leadership tasks. Surgeons with leadership roles, on the other hand, must manage patients and leadership tasks. Cooperation between the two occupational groups is vital, but limited access to surgeons frustrates nurses with leadership responsibilities in their day-to-day activities:

I believe it [work] can be structured in a better way. That is my claim, but I believe that a nurse leader has a different understanding of what leadership is than our surgeon counterparts. Work of a surgeon is more technical, and surgery is rated most important by leader surgeons. When I chose to become a leader for the nurses, I had to put aside day-to-day nursing of patients because it is not possible to be a good leader at the same time as nursing patients. On the other hand, I think if you are going to lead surgeons, then you need to excel in your profession to earn respect. Nurse with leadership responsibilities

VSD revealed that most employees are very loyal to the leadership in the hospital. Dialogue is nearly always with the staff member’s team leader. This aligns with the principles of VSD and contrasts favorably with high-level intervention into the daily operations. It was a key strength found in the Department of Orthopedic Surgery and one to build upon.

In addition, VSD found that exchanges in operational activities are extensive and widespread, demanding a well-functioning set of coordination procedures (next section). For example, medical staff move between operational activities. A surgeon transits between outpatients, surgery, and the wards. Surgeons specialize in types of surgery in part to increase overall efficiency. Surgeon resources are fixed, and so surgery is strongly impacted upon by their sick leave and such factors. Nurses may be deployed across operational activities to share scarce resources. In special cases, theatre nurses are moved between sub-specializations, though ward nurses tend to remain in their designated ward.

In summary, the operational arrangements are standardized across the hospital and are not fundamentally questioned by staff. There was no evidence of high-level intervention into the daily operations. VSD detected that the main organizational difficulties arose from support activities of the Department of Orthopedic Surgery – coordination, control, intelligence, and policy – rather than operational arrangements. Coordination turned out to be a major area of concern.

### Coordination

Coordination in the VSM is about the short-term distribution of resources and management of any conflict that arises therefrom. It involves procedures for the most favorable utilization of common resources in the short term. However, resource planning has limitations in a hospital department. The influx of patients, which determines the activity level, is predictable only to a limited extent and the medics must always be prepared to alter daily plans and to respond to sudden changes. The Department of Orthopedic Surgery has great need for the coordination of its activities; for optimal use of resources, efficient and effective handling of patients, and to ensure that crucial information about plans and patients are distributed to the right staff. The need for coordination is reinforced by the constant flow of resources, occupational groups, and patients between operational activities (Divisions).

VSD in the Department of Orthopedic Surgery found that staff have numerous regular coordination meetings. Many of the meetings are concerned with coordination of current and upcoming daily activities. For the morning shift, at 08:00, ward nurses with a leadership role and outpatient recovery staff come together to coordinate patient flow, availability of beds, and other resources. At 13.00, ward nurses come together for a daily coordination meeting to check on the status of activities and to make sure that the day’s tasks can be completed. Further, each ward designates a person to meet up with corresponding persons in the other wards, creating a communication channel with a focus on day-to-day resource coordination. Success relies on the staffs’ abilities to problem solve on the spot.

Surgeons, ward nurses and theatre nurses each have a daily morning meeting. The meetings serve as a communication link between the night and day shifts, and coordinate resources according to the known activity level for the day. The content of each meeting differs between the occupational groups, but the meetings were frequently mentioned by all staff involved as one of the most important activities for daily coordination and information flow.

In addition, a weekly meeting brings together an interdisciplinary team of surgeon leaders, nurse leaders, and representatives from administration. Meeting attendees include staff from outside the department but who are vital to its running, for example, anaesthetists. The purpose of the meeting is to review the working week and thereby adjust upcoming activities and short-term distribution of resources. Although this is a vital interdisciplinary meeting, it is often cancelled due to absences or other reasons:

We have operational meetings once a week with surgery – surgeons and surgeon leaders, outpatient clinic, outpatient recovery, the entire bunch. It is supposed to be once a week, but it was cancelled this week and last week. There have been many seminars for the surgeons; I believe that is why. Nurse with leadership responsibilities

We did have the operational meetings weekly. Nowadays they are every now and then, not regular anymore. We are summoned when necessary. It is sad because the meetings are an arena where surgery, anesthesia, outpatient clinic, the wards, and others have a chance to meet. Administrative staff

The ‘doctor’s round’ is the only formal meeting point between surgeons and ward nurses. It is extremely important for patient treatment because the ‘round’ is where surgeons receive information about each patient’s condition and based on this the surgeons give further instructions to the nurses. Ward nurses see the ‘round’ as the most important of all meetings but find it deficient:

They [the surgeons] usually do the rounds around 09.00, but after that we need someone to stay and talk to the nurses who take care of the patients, to let us discuss what we have seen. Sometimes no one stays after the rounds. Surgeons are in surgery or doing other things. Then nurses are left unable to do their job because of a lack of authorization. There are many decisions that must be made by surgeons that are left waiting. For example, this can lead to delayed discharge of patients because nurses lack the papers and the information from surgeons. Ward nurse

Nurses are highly dependent on instructions from surgeons and are forced to find alternative ways of communicating with them.

We meet the surgeons during the daily rounds. But after that, they are not easily available. We use the tools that we have. We use the operational plan to see when they are done with surgery and call them right away; nurses line up to do this. Nurse with leadership responsibilities

That said, surgeons do value the information obtained during the ‘rounds’ and agree that it is an important meeting point. A surgeon holding leadership responsibilities recognized that nurses observe patients throughout the day and are privy to important information:

I need information from the nurses in the wards about the patients. They observe the patients 24-h a day and the rounds are the only time during the day when they report on developments. Surgeon with leadership responsibilities

Department of Orthopedic Surgery has established a weekly meeting to discuss treatment of infected patients or patients at risk of infection, to better cope with the risk of infection. The meeting helps to increase interdisciplinary cooperation by drawing together wide-ranging expertise in decisions about patient-related activity. The importance of this coordination activity was recognized by one of the participating surgeons:

We have managed to find a time where many people are able to attend and it is a strength having so many representatives from each discipline, which is not common in hospitals. It is especially useful to be able to discuss the different cases and through the 4–5 years that we have had these meetings, I genuinely feel that they have raised quality and cooperation. Surgeon with leadership responsibilities

Nurses with leadership responsibilities spend much time ensuring that there are enough nurses to cover day-to-day activities. They also coordinate resources like bed capacity/availability for patients. Whole days are spent on such tasks. Nurses with leadership responsibilities were asked about the department’s greatest limitation.

That is the number of beds. It is a problem. We spend too much time finding beds for the patients. I don’t know how many people in this hospital in total spend their time on that, but all of the leaders at my level are working on bed allocation and it requires a lot of time. Nurse with leadership responsibilities

Cooperation between units and sections is key to ensuring that employees can do their job effectively. Surgeons and nurses with leadership responsibilities held different opinions about success in cooperation:

We have a great need to cooperate across the sections because many things are interdisciplinary, meaning that we need help from other subspecialists, and they need help from us. We need to discuss if the patient should stay in hospital and receive further interdisciplinary treatment. That is not functioning optimally, but not poorly either. Surgeon with leadership responsibilities

I feel that it is a challenge to cooperate with other occupational groups. I most often feel alone. I wish there was more interdisciplinary cooperation. As a leader and nurse in a clinic like this, you feel great pressure from the emergency room to get the patients to the right place at the right time. I feel that I am working against something. The surgeon resource is pressured as well; they have conflicting roles. However, I think that we do not feel the same pressure about discharging patients, in order to take in new ones. That is a challenge. Nurse with leadership responsibilities

A staffing center is the hub of the coordination activity. It aims to provide different units with nursing resources as and when needed. Units ‘lend’ resources to the pool of resources. Resources are distributed according to registered needs. However, the staffing center seldom has the required resources needed, for reasons evident above, especially in the short-term when the need is most pressing. Opinions varied considerably about the overall benefit of the center.

To sum up, there is a comprehensive meeting structure that aims to deal with issues of coordination, but the meetings do not deal with all essential issues and the key interdisciplinary meeting is often cancelled. There is potential to coordinate and employ resources more effectively with an improved and reliable meeting structure. Several focused questions should stimulate dialogue between stakeholders over this troublesome aspect of the department’s activity:

- Is there a way of improving communications between nurses and surgeons each day after the doctors’ round?
- What would it take to guarantee the weekly interdisciplinary meeting as it is the only arena in which to discuss challenges between cross-occupational groups and sections?
- Are there decisions that could be taken by nurses rather than surgeons to enable nurses to get on with their work?
- Is there a need for a better understanding of the difficulties that each occupational group faces by other occupational groups, to facilitate a more cooperative approach? There is some understanding and appreciation, but perhaps sessions could be arranged where nurses and surgeons are given the opportunity to elaborate on the problems of their occupation that they believe are not fully appreciated by other occupations.
- Is there a need to illuminate what nurses with leadership responsibility have to do to get work done? Few formal procedures exist to guide efforts to make daily operations run smoothly, e.g., the logistics of making sure that staff are in the right place at the right time. Rather, informal procedures are worked out separately by different leaders and carrying them out takes up much of their time that otherwise could be spent on management tasks.
- Can the staffing center be made more effective?

### Control

Control in the VSM focuses on longer-term issues than coordination. Planning is an integral part of any organization and the success of daily activities depends upon effective control aimed at meeting the plans. Control incorporates both resource bargaining and audit procedures.

VSD in the Department of Orthopedic Surgery identified three regular meeting points that contribute to control. First, there is a meeting every six months for long-term planning called the ‘activity planner’. The agenda is mainly to plan and to distribute surgery resources between operational activities. The process includes resource bargaining and reviews claims from the Divisions for increased resources for the coming period. Longer term issues are discussed too, for example, to do with anesthesia and the hospital’s plans. Representatives from elsewhere in the hospital may be invited to provide feedback and answers. An activity plan is created during the meeting and is made available to operational staff. The activity planner also acts as an information channel for intelligence activities leading to policy as it provides a ‘status summary’ on how the department is coping with demand (i.e., the strengths and weaknesses of the department). Second, each year the ward nurses with responsibilities travel together to a retreat in the country to discuss long-term challenges and to agree long-term plans. Third, an irregular interdisciplinary meeting is held by the head of the department where the leaders in the department meet and discuss longer-term topics.

Audit procedures are particularly vital to the viability of an organization where activity never ceases, and where decisions are made ‘on the go’. Some nurses wish that quality audit was better integrated into their everyday working life and more focused on the patient. Currently, staff are measured on cost, time usage, and other measures that relate to efficiency and economy rather than fundamental value-creating activities of quality of patient care. A theatre nurse explained how they are measured on time, but not the quality of patient care.

What we are monitored by is the time matrix. It says that you are supposed to use 45 min on preparations, and the surgical intervention should take two hours; and then there should be 20 min to finish up, wake up the patient and get him or her out of the recovery room. And 20 min for cleaning. And it will show as a Gantt chart and they [managers] will ask why an over-running activity took so long? We feel that we always must explain ourselves. First, we needed a catheter, we were not informed, and then we needed an extra anaesthetist, [and so on]. I am responsible for the time that I take to do things, not the quality of patient care. The time matrix puzzles me. Theatre nurse

Theatre nurses believe in quality in their work, but there is a lack of formal feedback on how they are performing and possible areas of improvement:

I feel that we have done a good job when the patient wakes up when he or she is supposed to, and everything is ok. The intervention went as planned, we know we did our jobs, but only we know that. I miss feedback because I am sure that there are [subsequent] infections and there may be cases related to other things that we do not know of. For as long as we do not hear anything, we are tempted to believe that everything is fine, but I know that that is not true. Theatre nurse

Interviews revealed that feedback available from audits does not always reach front-line staff:

We miss feedback on for example the infection register. Are our numbers high compared to the rest of the country? Those are the measures that the hospital receives and the clinics receive, higher up in the system than us, but which could be very valuable for us to see. But we must ask for it. It could be helpful to have for example monthly or quarterly feedback on how many infections we have had, how we compare to the rest of the country, and do we have any other deviations. Have the patients been placed in the wrong way on the operating table and gotten nerve damage? We never get this information because it is handled by administrators. Theatre nurse

Interviews revealed that quality is an important part of the surgeons’ profession. Surgeons co-evaluate their own work and work of other surgeons with the aim of further developing professional skills. However, there are no independent quality measurements:

We are to some degree measured on quality in what we do regarding how many infections we have and how many re-surgeries we have. We are measured on how long the waiting time is from when a hip fracture arrives until surgery. And we are measured on general waiting time for the emergency patients, ensuring that we get them through surgery within a reasonable time. And we are measured on how efficiently we run the surgery activity. Surgeon with leadership responsibilities

There are often situations where fluctuations in demand and activity level cannot be handled by increasing the number of resources through coordination or control procedures, and the only current solution is for staff to work even harder. Long-term lack of resources seriously affects the workforce and numerous personnel believe that this leads to high levels of sickness, causing a vicious spiral of under-resourcing. In practice, this problem is something that staff with operational and coordination duties must do their best to tackle on a day-to-day basis:

Nurses have the fundamental desire to help patients. That leaves us with no choice. We are left with the responsibility and need to make the best of all situations. Some shifts can be impossible, and you cooperate as best as you can with the people present and you can ask a leader or assistant leader to help if a crisis emerges. But at that point you have already reached a worrying stress level because you are already behind. You will always try to fix it yourself. Ward nurse

Some personnel are afraid that the department will become more and more pressurized due to a lack of resources making it harder to focus on the whole job because immediate needs become so urgent. A longer-term solution is required, and this points to the need for better intelligence (next section).

To sum up, a meeting structure exists that aims to achieve necessary control in the Department of Orthopedic Surgery and representatives from elsewhere in the hospital are invited to participate. However, two serious matters that arose are a shortage of personnel and weaknesses in quality audits. Two pertinent questions may help here:

- Does the department wish to influence centralized resource bargaining to achieve more effective resource allocation for the total hospital? How might the department raise this matter with hospital administrators?
- How can the department contribute to design and implementation of quality audit(s) that nurses desire with a focus on the fundamental value-creating activities of quality of patient care?

### Intelligence

Intelligence in VSM gathers and interprets information about the strengths and weaknesses of the organization and the opportunities and threats in the organization’s environment. The findings are formulated by procedures into useful information to support decision making about policy.

VSD found that one of the great strengths of the Department of Orthopedic Surgery is the unfailing commitment of the staff to the patient and to one another, even though resources are scarce and things sometimes ‘look dark’. One nurse stressed that no matter what the task or resource limitations, patients and colleagues remain central:

Your personal goal is that you really want to go home and feel that the patients that you have treated are happy. We always strive to do our best. And you really want to get done with everything during your shift; it is not a good feeling to leave many responsibilities to the next shift. Nurse

VSD found weaknesses in the link between the department’s activities and what is going on elsewhere in the hospital. One nurse with leadership responsibilities was under the impression that the intensity of day-to-day activities prevents a better-informed activity:

We are looking straight down at our papers and are focused on what we do, solving everyday problems and challenges. I do not feel that we have the time to look ahead; it is [we are] coping day-to-day. I miss having time to be able to look ahead and think more widely about the longer term. But there is so much to do that I do not have the time or capacity [to look ahead/think widely]. Nurse with leadership responsibilities

The department recently introduced a ‘monthly focus’ to address such problems. The focus is on important topics that either need improvement or are of great importance, e.g., work environment infections. The focus helps the department to work more systematically, in areas where it might otherwise struggle, based on intelligence gathered about operations.

The activity planner introduced above (under Control) also contributes to Intelligence. The head of the department provides a status report on how the department is performing compared to the demands and challenges that it is facing. Most of the intelligence data from day-to-day operations is gathered in statistics and for many areas it is possible to compare the department with other comparable departments, both inside and outside the hospital.

VSD found a need to emphasize a serious concern about a potentially damaging weakness in the department, the demoralization of staff, especially of the nurses. A subject that came up time and time again during the interviews is that patients are getting sicker in increasing numbers, with more complex diagnoses, while the resources for nurses are becoming ever scarcer. Interviews revealed that this worries staff, and many find the challenges daunting and steadily becoming insurmountable.

To sum up, intelligence gathering is rather weak, despite the introduction of a ‘monthly focus’ and contributions from the activity planner. As a result, one of the great strengths of the department has perhaps become one of its greatest weaknesses. The strong culture of the department and the wider hospital is good quality patient care. This powerful culture leads to an unfailing commitment of the staff. The concern, however, is that staff, in particular nurses it seems, will work themselves until sickness and stress stops them. There is inadequate awareness in the organization of the significance of these stress levels and little is done to address the problem.

Two questions need addressing:

- Nurses feel that they have no time to make informed improvements. How can this be problematized and overcome in the department?
- How can stress levels, in particular stress in the community of nurses, be made a top priority issue in the department that demands rectifying action?

### Policy

Policy in the VSM is responsible for mission, goals, objectives, values, and culture. Although higher-level management often performs parts of this function, with VSD neither the policy making, nor the intelligence gathering are considered merely a top-down hierarchical activity.

VSD in the Department of Orthopedic Surgery found that policy largely derives from outside. The hospital of concern is owned by the government and operated with the intention of fulfilling the local community’s need for quality health services. Many goals are given by the hospital or are national standards. However, as already seen, much of what drives the organization is a powerful organizational culture – the desire to conduct good patient treatment and pride in the occupation. Based on this, there is a strong positive attitude toward policy in the department, though it is let down to some extent by hindrances caused by weaknesses in Coordination and Intelligence already discussed. VSD did not surface any pressing questions specifically concerning policy but two generalized questions might be worth considering:

- Is policy effectively disseminated and understood throughout the department?
- Does policy adequately reflect the needs and capabilities of the department?

### General

**Fig. 4:**
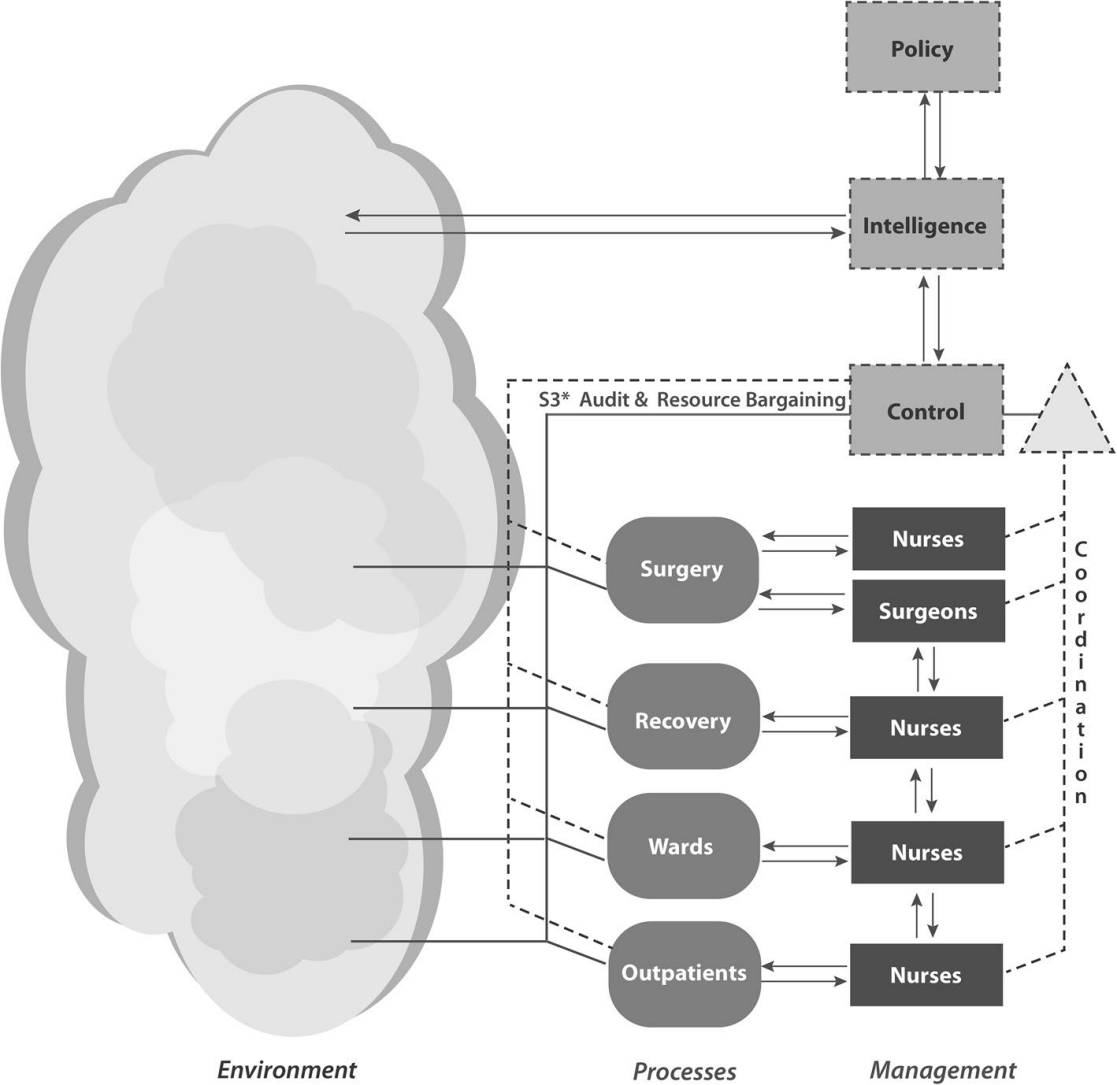
Deviations of orthopedic surgery clinic activities from the Viable System Model

Overall, VSD surfaced four main deviations from the principles of the VSM (Fig. 4).

1. Dotted rather than solid lines highlight where we found poor communication between management functions. Further, Coordination was particularly weak in this respect.
2. The dotted line around a management function indicates a weak management function. Control was particularly confused in this respect.
3. The dotted line from System 3* (S3*) indicates weaknesses in audit and monitoring.
4. Surgery was most problematic, with conflicting management activities of the nurses and surgeons, and a poor distinction in surgeon activities between Process and Management. Arguably, a continuing study should take Surgery as the System in Focus.

Although there are organizational weaknesses in the department, all management functions of the VSM are present and this supports organizational viability.

### Initial Response to Results, Reflections and Questions

The Projects Management Team recognized the above findings and could recall them from their own experiences. Several topics generated much discussion. One was the differences between the leadership of the surgeons and the nurses and how leadership might be combined into one function. Other popular topics were information flow between surgeons and nurses, the coordination of personnel and bed capacities, and how to create meeting arenas between the different disciplines. Discussions also raised greater awareness about how to introduce systemic thinking into the everyday work life of the Department of Orthopedic Surgery. The head of the clinic confessed that he had had worries in the beginning about what he saw as a rather complex model (the VSM), but that he was impressed with the findings that VSD surfaced. Clearly, VSD had generated an agenda that stimulated much debate and learning among the Projects Management Team and the many other stakeholders.

Subsequently, we presented the results to the doctors and researchers involved in the hospital’s center for developing and coordinating research and development projects. This group comprised some of the heads of clinics and project managers responsible for change projects within the hospital. The heads of the clinics were enthusiastic about VSD and its findings and commented that VSD had pinpointed problems that they recognized and, more importantly, the use of VSD had generated fresh insights into and enthusiasm for tackling these problems. Both doctors and researchers agreed that the top management of the hospital should have prioritized a research project using this approach for the entire hospital.

These first responses along with similar responses in member-check meetings were tangible evidence that our style of VSD had performed well as a problem structuring process. We planned for a follow up interview with the head of clinic in March 2020, but this has not been possible due to hospital effort concentrating on the Covid-19 pandemic. Unfortunately, at this point our brief with the hospital was suspended.

### Further notes on VSM and VSD

Smith and Shaw (2019) developed a framework representing characteristics of a Problem Structuring Method (PSM). They developed their framework from Rosenhead and Mingers (2001), who define PSM as a class of qualitative Operational Research (OR) modelling approaches designed to tackle ill-structured problems. Smith and Shaw (2019) do not accept that the VSM is such a PSM, primarily because their interpretation of the VSM does not appreciate ways in which the model can get to grips with participants’ subjective interpretations of the world. In other words, they do not hear Beer’s words about how modelling can, indeed should, involve the facilitation of participants and how the principles of participation enhance participants’ learning about the situation (e.g., Beer 1985, 1994). Indeed, Smith and Shaw (2019) appear to separate VSD from VSM, thus striping out the participative aspect of Beer’s wide-ranging contributions (e.g., see Team Syntegrity; Beer 1994).

Smith and Shaw’s (2019) interpretation of the VSM has also been criticised for being too narrow (e.g., Harwood, 2019). In essence, the criticism highlights a distinct difference between researchers who understand the VSM as a means by which to model organizational reality ‘that exists out there’, and researchers who adopt a constructivist approach, such as our hospital case study, that works in a fair way with the perceptions and understandings of involved and affected parties in a process of diagnosis and making improvements. This distinction is important when evaluating VSM and VSD. Indeed, VSM and VSD may be carried out through any one of the many participatory approaches exemplified by action research (e.g., Flood 2010; Heurich 2015; Lamé et al. 2017).

Further, Ackermann (2012) raised concern that participatory OR PSMs have been overlooked by many of the OR community. Two particularly persuasive explanations for this are: (1) Participatory approaches offer insights as the basis of decision making rather than testable results. (2) Managing content and managing processes at the same time is demanding, i.e., ‘working with’ rather than ‘on behalf’ of groups adds complexity. However, as evidenced from practice, there are significant benefits of ‘working with’ involved and affected parties in the process of VSD. Espinosa and Walker (2013), for example, chose a participative approach to deal with the ‘implementation gap’ (Atkinson 2006), i.e., scarcity of resources available to organizations seeking to align existing processes and structures with a new strategy. They used the VSM as a hermeneutical enabler, allowing the participants to share a common mapping of their organization, and this resulted in more effective organization and improved viability. A clearer mapping of the required tasks and roles shared by all allowed them to self-organise, basically because the VSM became a shared language for the organization which they reapplied continuously as new challenges appeared. There are numerous cases with similar benefits where VSD is supported by a participatory approach in the process of diagnosing, developing, and improving their future (e.g., Espinosa and Walker 2013; Tavella and Papadopoulos 2017).

Our approach to VSM and VSD that underpins the hospital case study reported herein is in line with participative and constructivist approaches. We argue for critical thinking that draws into the process people’s understanding and aspirations, recognizes and problematizes processes of power in organizational activities, and reflects on choices and ethical issues (Flood and Romm 1996; Flood and Finnestrand 2020). That said, what is it that is special about the VSM compared to other modelling approaches that makes it particularly useful in problem structuring?

The main benefit of VSD in tackling organizational problems in hospitals, indeed all applications in organizational contexts, is that it is particularly powerful in facilitating systemic and holistic diagnoses (Hoverstadt 2020). Lienhard and Legner (2014) illustrate why this is important. Medical departments in hospitals often build their own isolated software tools, resulting in silos with a low level of interoperability and redundant, disorganized, and inaccessible medical information. This undermines organizational viability in a complex organization like a hospital. VSD in IT-based management support and planning challenges the oft used top-down approaches that, “rely on almost complete models of the organizations, in the form of business process or domain models. Such linear thinking might be straightforward given a manager’s desire for control, but this approach is too rigid to cope with a complex, decentralised organizational design, like a hospital” (Lienhard and Legner 2014). In contrast, a participatory approach to VSD aims to develop more useful tools that enable local and organization-wide systemic needs.

It is also worthy of note that that the VSM has been used in conjunction with other organizational concepts, fads, and process tools (Hoverstadt 2020). This is prevalent within the hospital sector. Lamé et al. (2017) look at the first phases of a change program that aims at better integration of a hospital subsystem. They propose the methodological combination of VSM coupled with Kotter's (1996) ‘8 Steps for Leading Change’ to structure problems in a hospital's outpatient Chemotherapy Department. The main problems mirror the ones encountered in our hospital case study. Of particular concern, coordination between the Pharmacy and the Oncology Department was limited to sharing incomplete information which led to a highly variable workload from one day to another. Due to the article being published before the project was complete, it was too early to establish in what ways VSD had led to a more viable organization. However, the project had established a sense of urgency (Kotter’s step 1), created a guiding coalition (Kotter’s step 2; with the head of the Oncology Department, the head of the Pharmacy, and the head of the Cancer Division), established a vision for the future (Kotter’s step 3), and made an effort to communicate this change vision (step 4). The project had managed to create a collaboration between the Pharmacy and the Oncology Department, embodied in a series of work meetings, and this had already achieved improved coordination.

Love and Cooper (2010) carried out ‘a user-focused design analysis’ of in-hospital residential treatment for stroke patients in a dedicated stroke unit. They were able to focus on the user’s perspective of the hospital by following the progress of a single health service user, a stroke patient, observed from admission into the hospital to relocation three months later to a residential nursing home. Theoretically, Love and Cooper (2010) did this by triangulating the following three approaches. First, a focus on simple non-systemic design failures. Second, through the use of VSD. Third by reflecting on this through Ashby’s Law of Requisite Variety. VSD was employed to generate the first insights into everyday design failures or problems of the kind that can be addressed by individual, local redesign. VSD was prime in identifying structural systemic factors that caused problems in the stroke unit and, it was inferred, in the wider hospital and health service provision. In particular, Love and Cooper (2010) found control failures that put unnecessary pressure on coordination activities. A result of this was that control responsibilities collapsed into the Divisions, and Control thus failed to provide an integrated management of Divisions.

These are just a few examples of the application of VSM and in particular VSD in a hospital context. There are many other examples in many different contexts. For the interested reader, we recommend consulting the following recent cases in the health topics, medicine procurement systems (Mouhib et al. 2019) and health cluster development (Wong and Hiew 2019). An excellent broader overview to health systems research from a systemic point of view is given by Jackson and Sambo (2019). Much can be gleaned from a variety of other applications, such as viable organizations in disaster response (Preece et al. 2013, 2015), the development of local communities (Espinosa and Walker 2013; Espinosa and Duque 2018; Velásquez-Rodríguez and Payán-Durán 2021), learning and educational development (Hart and Paucar-Caceres 2016; Rezk and Gamal 2020), governance of IT and software projects (Arghand et al. 2021; Puche-Regaliza et al. 2020), and in tourism (Sánchez-García et al. 2020; Núñez-Ríos et al. 2020).

## Conclusion

As we learnt first-hand, a hospital is an extraordinarily complex form of organization. Most hospitals are large organizations that deal with a wide variety of illnesses and patient needs. The skill base required to provide the service is truly diverse, from specialist medical knowledge to advanced technical support, to modern approaches to administration. Hospitals bring together medics, technologists, and administrators, and the cultures that these professions bring with them. On top of this, an ever-increasing number of patients and scarcity of available resources threaten the viability of hospitals. Conceiving of effective arrangements for clinics and departments, and involving involved and affected stakeholders in the dialogical process, is the most challenging task that we have yet faced.

However, our case study demonstrates that the VSM and VSD can assist by striking at the heart of the hospital viability problem and by showing that maintaining viability requires an ability to communicate well and to be able to adapt to the complex ever-changing organizational context. VSD was an essential guide that led the process to recognize key information and insights about communication, interactions, and organizational patterns in the hospital. VSD helped by spotlighting organizational flaws and shortcomings from which we were able to construct an agenda for debate between key personnel that focussed on change and improvement. The findings generated through the process of VSD provided solid evidence for the debate process, ones that resonated with the experiences of involved personnel. The agenda for debate enabled visualization of the issues that were constraining viability (Espejo 2003; Espinosa and Walker 2011; Hildbrand and Bodhanya 2015). In other words, VSD facilitated investigation of the current organizational design by involved and affected stakeholders, offered a guide to improvement of the communication structures, and supported the process of change management (Espejo and Gill, 1997; Leonard and Beer 1994).

We offer our case study as an example of problem structuring facilitated by VSD. The case demonstrates that running a hospital service for current and future generations – keeping the blood flowing – can significantly benefit from such a systemic problem structuring approach. The case study also adds to the cumulative practical evidence that VSM and VSD are particularly suited to getting to grips with problems that arise in complex organizational settings.

## Data Availability

Not applicable
